# Draft Genome Sequence of Strain R13-38 from a Francisella tularensis Outbreak in Sweden

**DOI:** 10.1128/genomeA.01517-14

**Published:** 2015-02-19

**Authors:** Erik Alm, Abdolreza Advani, Andreas Bråve, Tara Wahab

**Affiliations:** Public Health Agency of Sweden, Department of Microbiology, Solna, Sweden

## Abstract

We have whole-genome sequenced a Francisella tularensis subsp. *holarctica* (also known as type B) strain from an outbreak in Sweden in 2013, derived from a privately owned well for drinking water.

## GENOME ANNOUNCEMENT

Francisella tularensis is a small, highly virulent, Gram-negative, nonmotile, nonsporulating, capsulated, and facultative intracellular pathogen which causes tularemia in humans and animals (1). This disease has been reported in Sweden since 1931, causing between 100 and 700 infections annually with no cyclical pattern or trends (2). Transmission occurs through arthropod bites (mosquitoes and ticks), inhalation of contaminated dust, and ingestion of contaminated food or water. Most of the cases are identified on the basis of clinical criteria and serology; isolation of bacteria is much less common. Many clinicians outside endemic areas fail to recognize the symptoms, and the real number of cases is probably higher than reported. The natural reservoir for tularemia is unclear, but there is an epidemiological relationship to water (3), and it has been shown that F. tularensis can grow and survive in Acanthamoeba castellanii (4), suggesting that protozoa are a significant environmental reservoir for F. tularensis. The pathogen is considered a potential biological weapon since aerosols of F. tularensis subsp. tularensis (also known as type A) are highly infectious and can cause life-threatening disease (5).

A local tularemia outbreak occurred in Sweden in 2013. The epidemiological investigation suggested that water from a privately owned well supplying a home with drinking water was the source of the outbreak. An isolate from the well (R13-38) was subsequently whole-genome sequenced, as this was the first instance where Francisella was isolated from water. One microgram of genomic DNA from strain R13-38 was used for Ion Torrent library construction on the AB Library Builder system using the Ion Xpress Plus fragment library kit. Lab Chip XT was used for library size selection at 400 bp and Lab Chip GX for quality control of the libraries. The libraries were enriched on beads using the OneTouch 2 system. The final template was sequenced in multiplex on an Ion 318 version 2 chip using the instructions from the manufacturer. The data were demultiplexed in Torrent Suite and analyzed in CLC Genomics Workbench version 7.5. The raw read data comprised 1,239,604 reads, and the average raw read length was 209 bp. The data set was *de novo* assembled and annotated using standard settings in CLC Genomics Workbench with the Genome Finishing Module version 1.4. This strain has a genome size of 1.74 Mbp with a GC content of 32.25%. The *N*_50_ contig length was 12,635 bp, with the longest contig being 38,598 bp; 241 contigs were longer than 500 bp. The average coverage for the contig sequences of strain R13-38 was 149×.

### Nucleotide sequence accession number.

This sequence belongs to NCBI Bioproject PRJNA269095 and was deposited at DDBJ/EMBL/GenBank under the accession number JUJV00000000.
